# Renal function and oxygenation are impaired early after liver transplantation despite hyperdynamic systemic circulation

**DOI:** 10.1186/s13054-017-1675-4

**Published:** 2017-04-11

**Authors:** Jenny Skytte Larsson, Gudrun Bragadottir, Bengt Redfors, Sven-Erik Ricksten

**Affiliations:** Department of Anesthesiology and Intensive Care Medicine, Institution of Clinical Science, Sahlgrenska Academy, University of Gothenburg, Sahlgrenska University Hospital, Blå Stråket 5, plan 5, 413 45 Gothenburg, Sweden

**Keywords:** Liver transplantation, Acute kidney injury, Renal blood flow, Glomerular filtration rate, Renal oxygen consumption, Renal oxygenation

## Abstract

**Background:**

Acute kidney injury (AKI) occurs frequently after liver transplantation and is associated with the development of chronic kidney disease and increased mortality. There is a lack of data on renal blood flow (RBF), oxygen consumption, glomerular filtration rate (GFR) and renal oxygenation, i.e. the renal oxygen supply/demand relationship, early after liver transplantation. Increased insight into the renal pathophysiology after liver transplantation is needed to improve the prevention and treatment of postoperative AKI. We have therefore studied renal hemodynamics, function and oxygenation early after liver transplantation in humans.

**Methods:**

Systemic hemodynamic and renal variables were measured during two 30-min periods in liver transplant recipients (*n* = 12) and post-cardiac surgery patients (controls, *n* = 73). RBF and GFR were measured by the renal vein retrograde thermodilution technique and by renal extraction of Cr-EDTA (= filtration fraction), respectively. Renal oxygenation was estimated from the renal oxygen extraction.

**Results:**

In the liver transplant group, GFR decreased by 40% (*p* < 0.05), compared to the preoperative value. Cardiac index and systemic vascular resistance index were 65% higher (*p* < 0.001) and 36% lower (*p* < 0.001), respectively, in the liver transplant recipients compared to the control group. GFR was 27% (*p* < 0.05) and filtration fraction 40% (*p* < 0.01) lower in the liver transplant group. Renal vascular resistance was 15% lower (*p* < 0.05) and RBF was 18% higher (*p* < 0.05) in liver transplant recipients, but the ratio between RBF and cardiac index was 27% lower (*p* < 0.001) among the liver-transplanted patients compared to the control group. Renal oxygen consumption and extraction were both higher in the liver transplants, 44% (*p* < 0.01) and 24% (*p* < 0.05) respectively.

**Conclusions:**

Despite the hyperdynamic systemic circulation and renal vasodilation, there is a severe decline in renal function directly after liver transplantation. This decline is accompanied by an impaired renal oxygenation, as the pronounced elevation of renal oxygen consumption is not met by a proportional increase in renal oxygen delivery. This information may provide new insights into renal pathophysiology as a basis for future strategies to prevent/treat AKI after liver transplantation.

**Trial registration:**

ClinicalTrials.gov, NCT02455115. Registered on 23 April 2015.

## Background

Acute kidney injury (AKI) is a common complication after liver transplantation, with a reported incidence of 11–57% [1–4]. Even a minimal increase in serum creatinine of 0.3 mg/dl (26.4 μmol/L) is associated with higher mortality and shorter graft survival after liver transplantation [5, 6]. Mortality after liver transplantation is reported to be 2–6% in patients not developing AKI, compared to a 47–55% mortality in patients who do develop AKI after liver transplantation [3, 4].

The etiology of AKI after liver transplantation is unknown, but is most likely multifactorial. Hypotension caused by intra-operative blood loss and reperfusion injury is presumably of importance. Furthermore, renal dysfunction may be present prior to transplantation as seen in patients with hepatorenal syndrome (HRS). In patients with HRS, a splanchnic vasodilatation is seen. This vasodilation is accompanied by an activation of the renin-angiotensin and of the sympathetic nervous system, resulting in increased renal vascular resistance. As a result, blood flow will be distributed away from the kidneys and hence the kidneys will receive a decreased oxygen delivery [7, 8]. This could be considered as a potential mechanism causing AKI after liver transplantation.

The diagnosis of AKI is based on an increase in serum creatinine according to Kidney Disease Improving Global Outcomes (KDIGO) criteria [9, 10]. Patients with hepatic failure usually have low levels of serum creatinine because of a low skeletal muscle mass, a lower creatine production and lower conversion of creatine to creatinine [11, 12]. Thus, creatinine-based methods for calculation of glomerular filtration rate (GFR) will overestimate measured GFR in this population.

To our knowledge, there is no data on the effects of liver transplantation on measured GFR, renal hemodynamics or renal oxygenation early after liver transplantation. Thus, to improve the prevention and treatment of postoperative AKI, it is of great importance to get more insights into the renal pathophysiology after liver transplantation. Indeed, in the most recent practice-based recommendations from the American Society of Transplantation Liver and Intestine Community of Practice, Levitsky et al. stress that nephro-protective strategies are needed to improve renal outcome after liver transplantation [13].

In the present study, we measured GFR, renal blood flow, renal oxygen consumption and renal oxygenation early after liver transplantation by using the retrograde renal vein thermodilution technique and by measuring the renal extraction of the filtration marker chromium ethylenediaminetetraaceticacid (^51^Cr-EDTA). Patients undergoing uneventful major cardiac surgery served as controls. We believe that the comparison between these two groups is relevant since both groups have been exposed to major surgery, both groups have had contact with material that is not endogenous, with a consequent systemic inflammation. Furthermore, both groups were sedated and mechanically ventilated during the experimental procedure, which was performed early after arrival in the intensive care unit (ICU). Hence, differences between the two groups with respect to renal function can be viewed as results-specific for liver recipients. Our primary end point was the change in measured GFR from baseline after liver transplantation. Our null hypothesis was that measured GFR is not affected in the immediate postoperative period after liver transplantation.

## Methods

The Gothenburg Regional Ethics Committee approved the study protocol and written informed consent was obtained from all patients within the 24 hours before surgery. The group of liver-transplanted patients was compared to a group of post-cardiac surgery patients after uncomplicated cardiac surgery, at a numerical ratio of 1:6. Patients in both groups were studied in the early postoperative period in the intensive care unit during sedation and mechanical ventilation.

### Liver transplant recipients

Twelve adult patients undergoing liver transplantation were prospectively included during the period of January 2015 to February 2016 with the following inclusion criteria: (a) age >18 years and (b) measured GFR > 60 ml/min. The exclusion criteria were: (a) intra-operative need for veno-venous bypass, (b) clinically significant postoperative bleeding, (c) unsuccessful catheterization of the renal vein, and (d) contraindication to radio-contrast agents. In all patients, GFR was measured within 3 months prior to transplantation by the plasma clearance of either ^51^Cr-EDTA or iohexol.

Anesthesia was induced by propofol and fentanyl or remifentanil, and maintained with sevoflurane and either of the opiates used for induction. Intra-operative blood salvage was performed with the Cell Saver® 5+ device (Haemonetics Corporation, Braintree, MA, USA). Packed red blood cells were given to maintain hemoglobin ≥ 80 g/liter and plasma and blood platelets were administered at the discretion of the attending anesthesiologist. Immediately before reperfusion, all patients obtained methylprednisolone at a dose of 0.5–1 gram and mannitol at a dose of 200–300 ml. A bolus dose of epinephrine (0.01–0.15 mg) was administered in reperfusion-induced hemodynamic instability. Norepinephrine was administered intra-operatively to maintain a mean arterial pressure > 65 mmHg.

On arrival to the intensive care unit (ICU), the patients were mechanically ventilated and sedated with propofol (2.7 ± 0.6 mg/kg/h) and either fentanyl or remifentanil. Postoperative targets were pulse pressure variation < 12% and a mean arterial pressure of 70–80 mmHg. Postoperative hypovolemia was treated according to routine clinical practice with albumin (Albumin Baxalta® 200 g/l) and/or crystalloid fluid (Ringer–Acetate®, Baxter Viaflo, Baxter Healthcare Corporation, Irvine, CA, USA). Hypotensive normovolemic patients were treated with norepinephrine according to the attending intensivist.

### Cardiac surgery group (control group)

Seventy-three post-cardiac surgery patients served as controls. These patients participated in pharmacological intervention trials performed by our research group in 2006–2014 [14–17]. The inclusion criteria were: (a) age >18 years, (b) elective cardiac surgery with cardiopulmonary bypass, (c) preoperative left ventricular ejection fraction ≥ 40%, and (d) preoperative serum creatinine within normal range. The exclusion criteria were: (a) postoperative need for inotropic support, (b) postoperative arrhythmias requiring treatment, (c) significant postoperative bleeding, (d) unsuccessful catheterization of the renal vein, and (e) postoperative AKI according to the AKIN criteria [18]. The baseline renal and systemic data of these patients, i.e. before pharmacological intervention, were used for comparison with those of the liver-transplanted group. Preoperative estimated glomerular filtration rate was calculated using the Modification of Diet in Renal Disease (MDRD) formula in all patients.

Anesthesia was induced by fentanyl and propofol. Before and after cardiopulmonary bypass, anesthesia was maintained with sevoflurane. During cardiopulmonary bypass, anesthesia was maintained with propofol. In the intensive care unit, the patients were sedated with propofol (3.8 ± 0.18 mg/kg/min) and morphine or fentanyl, and mechanically ventilated. Target central venous pressure (CVP) and target mean arterial pressure (MAP) were 5–10 mmHg and 70–80 mmHg, respectively. Postoperative hypovolemia was treated according to routine clinical practice with hydroxethyl starch (Venofundin, Braun, Germany) and crystalloid fluids (Ringer–Acetate®, Baxter Viaflo).

### Measurements of systemic hemodynamics

Arterial blood pressure was measured continuously via a femoral or radial artery catheter. CVP was measured continuously via a central venous catheter inserted through the right jugular vein. Cardiac output (CO) was measured by the transthoracic thermodilution pulse contour technique using the PiCCO™ device (Pulsion Ltd, Munich, Germany) in the liver-transplanted group, and in the cardiac surgery group by a pulmonary artery thermodilution catheter (Baxter Healthcare Corporation, Irvine, CA, USA). CO was measured in triplicate and indexed to the body surface area. Systemic vascular resistance index (SVRI) and stroke volume index (SVI) were calculated according to standard formulae. In the liver transplant recipients, cardiac index was recorded before, during and after the anhepatic phase.

### Measurements of renal variables

An 8 Fr catheter (Webster Laboratories, Baldwin Park, CA, USA) was postoperatively introduced into the left or right renal vein, via the right femoral vein, under fluoroscopic guidance. The catheter was placed in the central portion of the renal vein, the position being confirmed by venography using ultra-low doses of iohexol, 5–15 mg I kg − 1 (Omnipaque 300 mg I ml − 1; GE Healthcare, Stockholm, Sweden). Renal blood flow (RBF) was measured in triplicate by the continuous retrograde thermodilution technique [14, 17, 19, 20]. After the collection of blood and urine blanks, an intravenous priming dose of ^51^Cr-EDTA (GE Healthcare, Amersham, UK) was given, followed by an infusion at a constant rate, individualized to body surface area and to preoperative serum creatinine. Serum ^51^Cr-EDTA activity from arterial and renal vein blood was measured using a well counter (Wizard 3’, 1480, Automatic gamma counter; PerkinElmer LAS, Turku, Finland). Formulae for calculation of renal variables are described in Table 1. All renal data were normalized to a body surface area of 1.73 m^2^. Serum creatinine was measured in all patients within 24 hours before surgery and on the first and second postoperative days. In addition, serum creatinine was measured on admission to the ICU in the liver recipients.

**Table 1:** Formulae for calculation of renal variables

Variable	Formulae
Renal blood flow (RBF)	(Unilateral renal vein blood flow × 2) + urine flow
Renal plasma flow (RPF)	RBF × (1 – hematocrit)
Filtration fraction (FF)	(RPF × [^51^Cr-EDTA arterial] – (RPF – urine flow) × [^51^Cr-EDTA renal vein])/(RPF × [^51^Cr-EDTA arterial])
Glomerular filtration rate (GFR)	FF x RPF
Renal vascular resistance (RVR)	(MAP-CVP)/RBF
Arterial-renal vein (rv) oxygen content difference (RAVO_2_-diff)	(CaO_2_-CrvO_2_)
Renal oxygen consumption (RVO_2_)	RBF × (CaO_2_-CrvO_2_)
Renal oxygen extraction	(CaO_2_-CrvO_2_/CaO_2_)
Renal sodium filtration	GFR × [Na^+^]_s_
Renal sodium excretion	Urine flow × [Na^+^]_s_
Renal sodium reabsorption	(GFR × [Na^+^]_s_) – (urine flow × [Na^+^]_u_)

^*51*^ *Cr-EDTA, c*hromium ethylenediaminetetraaceticacid, *MAP* mean arterial pressure, *CVP* central venous pressure, *CaO*_*2*_ arterial oxygen content, CrvO_2_ renal vein oxygen content, *[Na* ^*+*^ *]*_*s*_ serum sodium concentration, *[Na* ^*+*^ *]*_*u*_ urine sodium concentration

### Experimental procedure

After an equilibration period of at least 60 min, two 30-min urine collection control periods were started. Thermodilution measurements of RBF and measurements of cardiac index (CI) were conducted at the end of each of the urine collection periods followed by blood and urine sampling. Infusion rates of fluids and of norepinephrine (liver-transplanted group) were not changed during the experimental procedure.

### Statistical analysis

Data on renal and systemic hemodynamic variables from the two 30-min measurement periods were pooled. The primary end-point of the present study was the change in GFR after liver transplantation compared to the preoperatively measured GFR. To detect a fall in GFR by 30%, ten patients were needed at a power of 0.80, a significance level of 0.05 and a standard deviation of 20 ml/min. Continuous variables were checked for normal distribution. Intergroup differences where compared using independent-samples *t* test or Mann-Whitney *U* test when appropriate. Categorical data were compared using Fisher’s exact test. Linear regression analyses were performed to correlate renal oxygen consumption to renal sodium reabsorption and GFR, respectively. PASW Statistics Version 18.0 (SPSS Inc., Chicago, IL, USA) was used for statistical analyses. Within- and inter-group repeated measurements of serum creatinine were calculated using mixed model in SAS (SAS version 9.3, SAS Institute Inc., Cary, NC, USA). An unstructured covariance structure was assumed for the inter-group analysis.

Data are presented as mean ± SD throughout the text. A *p* value < 0.05 was considered significant.

## Results

### Liver transplant recipients

In the liver-transplanted group, informed consent was obtained from 14 patients. One patient was excluded because of postoperative bleeding and another patient was excluded because of unsuccessful placement of the renal vein catheter. Individual preoperative and intra-operative data on the liver-transplanted group are shown in Tables 2 and 3, respectively. Primary sclerotic cholangitis was the most common liver-related diagnosis, followed by cirrhosis due to viral infection. The mean Model For End-Stage Liver Disease (MELD) and Child-Pugh scores were 14.0 ± 5.7 and 9.3 ± 1.7, respectively. Mean preoperative measured GFR (mGFR), estimated GFR (eGFR) and serum creatinine were 85.5 ± 18.7 ml/min, 86.9 ± 19.8 ml/min and 70.8 ± 13.9 μmol/l, respectively. The duration of the surgical procedure was 5.9 ± 1.4 hours and mean intra-operative bleeding was 2.3 ± 1.3 liters.

**Table 2:** Preoperative individual data, liver transplant recipients

Patient number	Diagnosis	MELD score	Child-Pugh score	Serum bilirubin (mmol/l)	mGFR (ml/min/1.73 m^2^)	Serum creatinine (μmol/l)	ASA
1	Primary biliary cirrhosis	15	10	36	75	67	3
2	Hepatitis C virus, cirrhosis	17	10	32	95	109	2
3	Primary sclerosing cholanigitis	6	9	8	72	60	2
4	Primary sclerosing cholanigitis, cirrhosis	22	11	110	62	59	3
5	Hepatitis C virus, hepatocellular carcinoma, cirrhosis	8	7	10	105	74	2
6	Alpha-1 antitrypsin deficiency, cirrhosis	16	11	43	94	75	3
7	Alcoholic liver cirrhosis	24	12	150	72	62	3
8	Primary sclerosing cholanigitis, cirrhosis	13	9	60	102	73	2
9	Primary sclerosing cholanigitis, cirrhosis	9	6	32	89	76	2
10	Hepatitis C virus, hepatocellular carcinoma, cirrhosis	9	9	14	122	60	3
11	Primary sclerosing cholanigitis, hepatitis B virus	18	10	340	77	60	2
12	Hepatitis C virus, hepatocellular carcinoma, cirrhosis	11	8	24	61	75	3
Mean		14.0	9.3	71.6	85.5	70.8	2.50
SD		5.7	1.7	94.6	18.7	13.9	0.52

Data presented as mean ± SD

*MELD* Model For End-Stage Liver Disease, *mGFR* measured glomerular filtration rate, *ASA* American Society of Anesthesiologists

**Table 3:** Intra-operative data of liver transplant recipients

Duration of surgery (min)	357 ± 86
Duration of anhepatic phase (min)	73 ± 30
Bleeding (ml)	2270 ± 1290
Packed red blood cells (units)	2.5 (0–4)
Platelets (units)	2.5 (0–4)
Plasma (units)	4.0 (0–6)
Fibrinogen (g)	2.0 (0–11)
Albumin (ml)	300 (0–600)
Cellsaver (ml)	400 (0–980)
Intra-operative crystalloid (L)	2.5 ± 0.9
Epinephrine, n (%)	9 (75)
Diuresis (ml/procedure hour)	118 ± 47
CI prior to anhepatic phase (l/min/m^2^)	3.3 ± 0.9
CI during anhepatic phase (l/min/m^2^)	3.9 ± 1.6
CI after reperfusion (l/min/m^2^)	4.4 ± 0.9
SVRI (ml/min/min^2^)	1413 ± 290
CVP (mmHg)	8 ± 2
Intra-operative fluid balance (ml)	665 ± 1540

Data are presented as median (min-max), mean ± SD, n = number of patients (%)

*CI* cardiac index, *SVRI* systemic vascular resistance index, *CVP* central venous pressure

### Liver transplant recipients versus post-cardiac surgery (control) group

Data on the characteristics of the two study groups are shown in Table 4. In the liver-transplanted group, the proportion of female gender was higher, the patients were younger, and they had a lower preoperative serum creatinine compared to the post-cardiac surgery group. The difference in preoperative estimated GFR between the groups was not statistically significant. Hypertension and treatment with angiotensin-converting enzyme inhibitor (ACE inhibitor) were less frequent in liver transplant recipients. A greater proportion of patients in the liver-transplanted group were treated with diuretics, while there were no statistical differences between the groups with respect to the use of beta-adrenergic blockers or calcium channel antagonists.

**Table 4:** Patient characteristics

Variable	Control group (*n* = 73)	Liver transplant recipients (*n* = 12)	*p* value
Gender, n (% female)	8 (11)	7 (58)	0.001
Age, mean (SD)	66.6 ± 10.1	56.7 ± 10.7	0.005
Body surface area (m2)	1.96 ± 0.2	1.92 ± 0.2	0.509
Body mass index (kg/m2)	26.4 ± 3.4	21.9 ± 1.1	<0.001
Hypertension, n (%)	40 (54.8)	1 (8.3)	0.004
Diabetes, n (%)	3 (4.1)	1 (8.3)	0.462
Beta-adrenergic blocker, n (%)	58 (79.5)	7 (58.3)	0.143
ACE inhibitor, n (%)	37 (50.7)	2 (16.7)	0.033
Calcium antagonist, n (%)	12 (16.4)	0 (0)	0.201
Diuretics, n (%)	3 (4.1)	8 (66.7)	<0.001
- Aldosterone antagonists, n (%)		6 (50%)	
Preoperative serum creatinine (μmol/l)	82.7 ± 11.4	70.8 ± 13.9	0.001
Preoperative estimated GFR (mL/min)	84.5 ± 14.5	86.9 ± 19.8	0.690
Preoperative measured GFR (mL/min)	-	85.5 ± 18.7	-

Values are means ± SD, n = number of patients (%). Estimated GFR; using MDRD (Modification of Diet in Renal Disease) formula

*ACE inhibitor* angiotensin-converting enzyme inhibitor, *GFR* glomerular filtration rate

### Systemic variables (Table **5**)

All liver-transplanted patients required norepinephrine infusion at a mean dose of 0.28 ± 0.17 μg/kg/min to maintain a MAP of between 70 and 80 mmHg. There were no statistically significant differences between the liver-transplanted group and the control group regarding MAP, heart rate, CVP, serum hemoglobin or systemic oxygen consumption index. CI (65%), SVI (69%) and systemic oxygen delivery index (60%) and venous saturation were all significantly higher (all *p* < 0.001), while SVRI was significantly lower (−36%, *p* < 0.001), in the liver-transplanted group compared to the control group. In liver transplant recipients, CI increased by 33% after reperfusion (*p* < 0.01) (Table 3).

**Table 5:** Systemic data in the immediate postoperative period

Variable	Control group (*n* = 73)	Liver transplant recipients (*n* = 12)	*p* value
Mean arterial pressure (mmHg)	74.0 ± 8.7	75.1 ± 1.1	0.338
Cardiac index (l/min/m^2^)	2.6 ± 0.5	4.3 ± 1.0	<0.001
Heart rate (beats/min)	76 ± 11	73 ± 17	0.522
Stroke volume index (ml/m^2^)	35.4 ± 7.1	60.0 ± 12.0	<0.001
Central venous pressure (mmHg)	7.9 ± 2.5	7.4 ± 3.0	0.639
Systemic vascular resistance index (dynes x sec/cm^3^/m^2^)	2081 ± 577	1337 ± 392	<0.001
Mixed/central venous oxygen saturation (%)	72.5 ± 4.4	81.2 ± 6.7	0.001
Serum hemoglobin (g/l)	106.2 ± 13	104.7 ± 15	0.730
Systemic oxygen delivery index (ml/min/min2)	381 ± 74	608 ± 142	<0.001
Systemic oxygen consumption index (ml/min/min2)	100.1 ± 15.1	94.5 ± 19.3	0.357

Data are presented as mean ± SD

### Renal variables (Figs. 1, 2, 3 and Table 6)

**Fig. 1:**
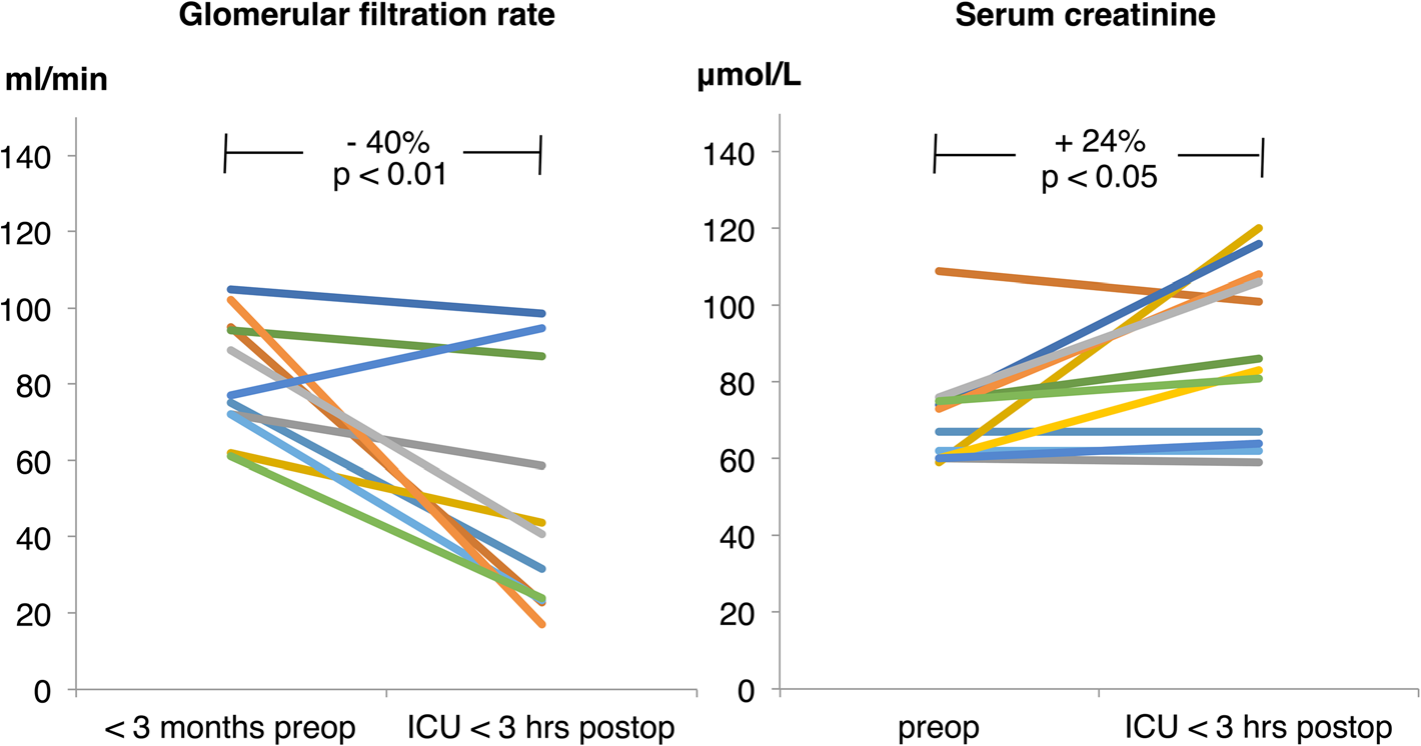
Individual data on measured glomerular filtration rate (GFR) and serum creatinine before (preoperative) and early after liver transplantation (intensive care unit [*ICU*] ≤ 3 hours) (*n* = 12)

**Fig. 2:**
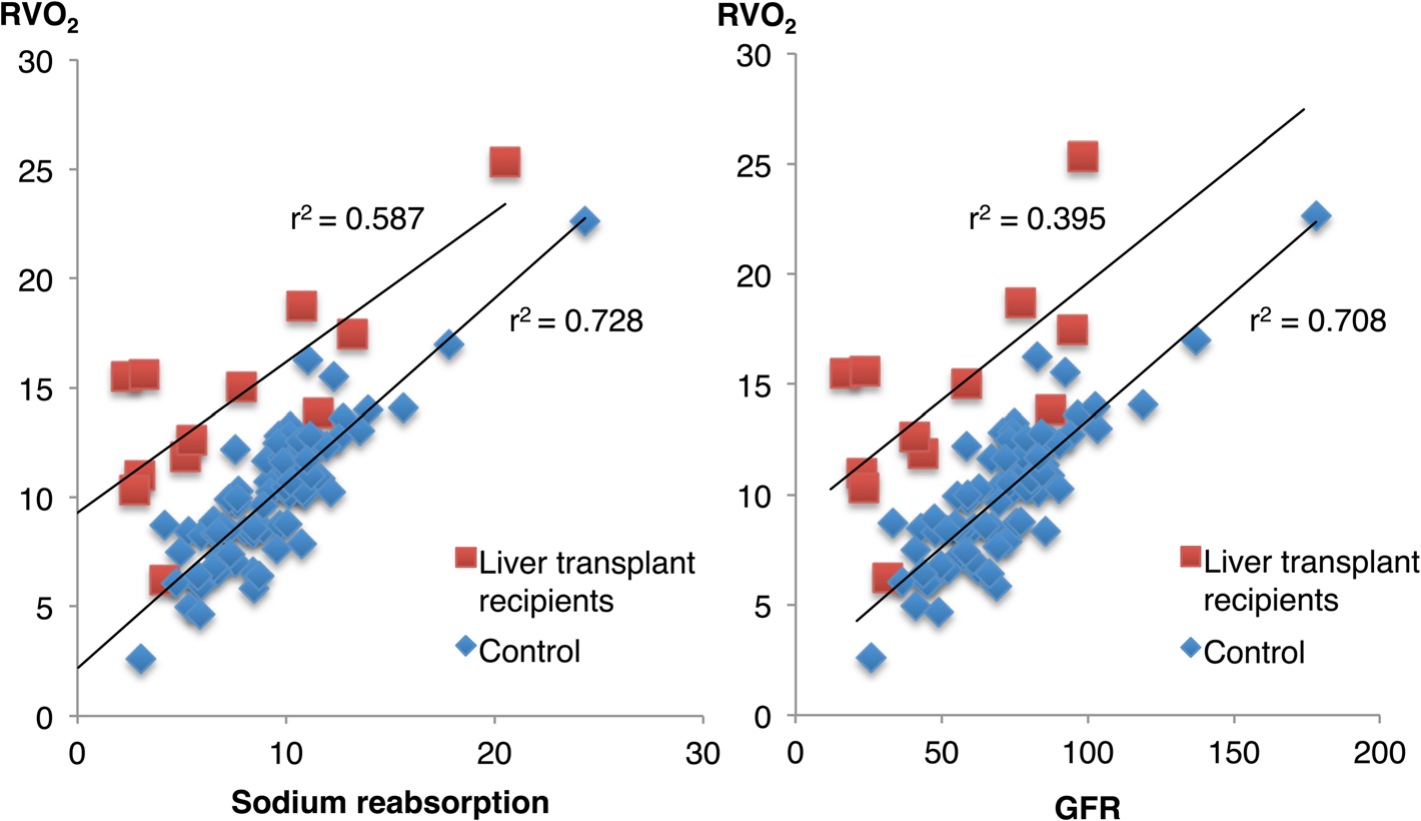
Relationship between renal oxygen consumption (*RVO*_*2*_) and renal sodium reabsorption, and between renal oxygen consumption and glomerular filtration rate (*GFR*), in the early postoperative period in liver recipients and after uncomplicated cardiac surgery (controls)

**Fig. 3:**
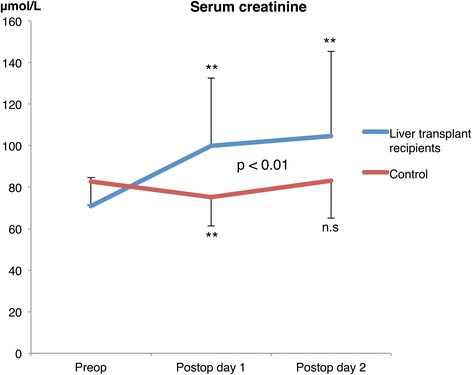
Changes in serum creatinine in liver recipients and after uncomplicated cardiac surgery (controls). **p < 0.01

**Table 6:** Renal data in the immediate postoperative period

Variable	Control group (*n* = 73)	Liver transplant recipients (*n* = 12)	*p* value
Renal oxygen extraction	0.100 ± 0.03	0.124 ± 0.04	0.042
Urine flow (ml/min)	3.13 ± 1.7	2.54 ± 2.2	0.065
Renal blood flow (ml/min)	716 ± 209	843 ± 197	0.024
Renal blood flow/cardiac index	0.277 ± 0.08	0.202 ± 0.05	<0.001
Renal vascular resistance (mmHg/ml/min)	0.100 ± 0.03	0.085 ± 0.02	0.051
Glomerular filtration rate (ml/min)	70.9 ± 23.3	51.5 ± 30.4	0.043
Filtration fraction	0.15 ± 0.04	0.09 ± 0.06	0.006
Renal sodium filtration (mmol/min)	9.67 ± 3.2	7.63 ± 5.6	0.052
Renal sodium reabsorption (mmol/min)	9.28 ± 3.2	7. 50 ± 5.5	0.065
Fractional sodium excretion (%)	5.0 ± 4.0	2.5 ± 3.5	0.002
Renal oxygen delivery (ml/min)	103.5 ± 32.1	119.7 ± 29.7	0.048
Renal oxygen consumption (ml/min)	10.0 ± 3.2	14.4 ± 4.8	0.001
Renal oxygen consumption/renal sodium reabsorption (ml/mmol)	1.15 ± 0.3	3.13 ± 2.4	<0.001
Serum creatinine day 1 (μmol/l)	75.1 ± 13.9	99.8 ± 32.7	0.025

Data are presented as mean ± SD

In the immediate postoperative period, mGFR decreased from 85.5 ± 18.7 to 51.5 ± 30.4 (−40%, *p* < 0.01) in liver transplant recipients. This decline in mGFR measured directly after liver transplantation was accompanied by a statistically significant increase in serum creatinine by 24%, from 70.8 ± 13.9 to 87.7 ± 18.7 μmol/l (*p* < 0.05) (Fig. 1).

RBF was higher (18%, *p* < 0.05) and renal vascular resistance was lower (15%, *p* = 0.051) in liver transplant recipients compared to the control group. The ratio between RBF and CI (RBF/CI) was 27% lower (*p* < 0.001) in the liver-transplanted compared to the control group. GFR and filtration fraction were 27% (*p* < 0.05) and 40% (*p* < 0.01) lower in liver transplant recipients. Renal oxygen consumption was 44% higher (*p* < 0.001) in liver transplant recipients despite a 19% lower renal sodium reabsorption compared to the control group. In both groups, there was a close correlation between renal oxygen consumption and renal sodium reabsorption, (control group: *r* ^2^ = 0.728, *p* <0.001, liver recipients: *r* ^2^ = 0.587 *p* < 0.05), and between renal oxygen consumption and GFR (control group: *r* ^2^ = 0.708, *p* < 0.001, liver recipients: *r* ^2^ = 0.395, *p* <0.05) (Fig. 2). However, the renal oxygen consumption per mmol/min of reabsorbed sodium was 2.7 times higher in the liver-transplanted compared to the control group. This increase in renal oxygen consumption was not met by a proportional increase in renal oxygen delivery, as demonstrated by the higher renal oxygen extraction in liver transplant recipients compared to the control group (*p* < 0.05). Serum creatinine increased in the liver-transplanted group by 41% (*p* <0.01) and 48% (*p* <0.01) on the first and second postoperative day, respectively, compared to the preoperative baseline value. This was not seen in the control group, and as shown in Fig. 3, there was a statistically significant difference in serum creatinine between the groups over time. Eight patients in the liver-transplanted group (67%) developed acute kidney injury, as defined by the KDIGO criteria, during the two first postoperative days.

## Discussion

The main finding of the present study was that there was an early and substantial decline in renal function after liver transplantation. In spite of this decline in GFR, and the lower renal sodium reabsorption, renal oxygen consumption was considerably elevated in liver recipients compared to after uncomplicated cardiac surgery. Furthermore, the renal oxygen supply/demand relationship (i.e. oxygenation) was impaired in liver transplant recipients, as the increase in renal oxygen consumption was not met by a proportional increase in renal oxygen delivery, despite the hyperdynamic circulation seen in this group.

The physiological control of GFR is mediated by the balance between the tone of the afferent and efferent arterioles. A fall in GFR may be caused either by vasoconstriction of the afferent arterioles, with reduced RBF as a consequence, or a vasodilation of the efferent arterioles, which will be accompanied by an increase in RBF. An activation of the tubulo-glomerular feedback mechanism, caused by tubular dysfunction, would induce an afferent arteriolar vasoconstriction with a decrease in both RBF and GFR [21, 22]. Our findings of a fall in GFR combined with an increased RBF, is thus best explained by vasodilation preferentially of the efferent arterioles. Another tentative explanation to the fall in GFR after liver transplantation, as an alternative to the “vascular abnormality” described above, would be tubular cell dysfunction (“tubular abnormality”) manifested as a decrease in tubular sodium reabsorption. This would increase the sodium delivery to the macula densa, activating the tubulo-glomerular feedback mechanism causing an afferent arteriolar vasoconstriction with a decrease in both RBF and GFR [21, 22]. This mechanism, however, is unlikely to explain the fall in GFR after liver transplantation, as renal vascular resistance in the present study was lower compared to the control group. Furthermore, we find the early fall in GFR in this study unlikely to be caused by shedding of tubular cells causing tubular obstruction, as this has been described to occur only in later phases of ischemic AKI [22].

Our group has repeatedly shown that there is a close correlation between GFR, tubular sodium reabsorption and renal oxygen consumption in postoperative patients [14, 19, 23, 24]. This was also demonstrated in the present study in patients early after liver transplantation (Fig. 2). The major difference between liver recipients and the control group was that renal oxygen consumption was higher at a certain level of tubular sodium reabsorption or GFR in the liver transplant group. This is demonstrated by the upward displacement of the curves relating renal oxygen consumption to tubular sodium reabsorption and GFR in Fig 2. Furthermore, the renal oxygen consumption per millimole reabsorbed sodium was 2.7 times higher in the liver-transplanted group compared to the control group. This could either be caused by tubular injury and an energy-inefficient tubular sodium transport, as demonstrated by our group in patients with early ischemic AKI [24], or by an increase in oxygen demand for basal renal metabolism. Extrapolation of the regression lines in Fig. 2 to the y-axis indicates the renal oxygen consumption in a non-filtering, non-reabsorbing kidney, i.e. the basal renal metabolism. Thus, the major explanation for the higher renal oxygen consumption after liver transplantation, despite the lower GFR and sodium reabsorption, seems to be an elevation of basal renal oxygen requirements. The mechanism behind this finding is, so far, unclear. It is not likely to be explained by a generalized increase in organ oxygen consumption, as the systemic oxygen consumption index did not differ between groups. One could speculate that the production and release of reactive oxygen species (ROS) from the liver graft, as a consequence of the ischemia/reperfusion injury [25], may contribute to the increased renal oxygen consumption, as it has been shown that oxidative stress increases mitochondrial oxygen consumption [26].

The renal oxygen/supply demand relationship, i.e. renal oxygenation, was impaired early after liver transplantation, expressed as the higher renal oxygen extraction compared to the control group. It is reasonable to assume that this impairment in renal oxygenation may induce tubular injury in the postoperative period, particularly so in the renal medulla, which is sensitive to ischemia. Medullary tissue oxygen tension is low already under normal conditions, because of the high oxygen utilization of the medullary thick ascending limb [27]. It has been shown that tubular injury markers, such as neutrophil gelatinase-associated lipocalin, are released in the urine within hours after liver transplantation and that this may predict AKI [28–30]. An early release of tubular injury markers early after liver transplantation could be explained by the impaired renal oxygenation demonstrated in the present study.

In this group of patients, with advanced or moderately advanced chronic liver disease, a hyperdynamic circulation was seen, with a profound systemic vasodilation and a high cardiac index. Such a hyperdynamic circulation in patients with advanced cirrhosis has been suggested to be caused by a splanchnic vasodilation [31] in turn caused by augmented levels of nitric oxide [32]. Furthermore, there is a post-reperfusion increase in cytokines and complement factors, which also contributes to the systemic vasodilation resulting in a perioperative need for vasopressor treatment during liver transplantation [25]. In the present study, the hyperdynamic circulation was accompanied by a redistribution of RBF away from the kidneys, as illustrated by the 27% lower RBF to CI ratio. The systemic vasodilation in the liver-transplanted group was treated with norepinephrine. As norepinephrine has been shown to decrease renal blood flow in volunteers [33, 34], one could argue that this might have mitigated an even more profound renal vasodilation and renal hyperemia that would otherwise have occurred. We believe that this is less likely, as we have shown that restoration of mean arterial pressure from 60 to 75 mmHg by increasing the dose of norepinephrine, improves renal oxygen delivery, GFR and renal oxygenation in postoperative patients with norepinephrine-dependent systemic vasodilation and AKI [35].

An alternative approach to treat systemic vasodilation during and after liver transplantation would be to use the vasopressin-analogue terlipressin, which is metabolized to vasopressin. In a randomized, controlled study, Mukhtar et al. studied the effects of terlipressin versus saline on splanchnic hemodynamics and postoperative renal function in patients undergoing liver transplantation [36]. Terlipressin improved renal function, as serum levels of creatinine and cystatin C were significantly lower in the terlipressin group during the two first postoperative days. Bragadottir et al. analysed in a pharmacodynamic study the renal effects of vasopressin in uncomplicated post-cardiac surgery patients and found that vasopressin induces a vasoconstriction of the efferent arterioles causing an increase in GFR and renal oxygen consumption but a decrease in renal blood flow [14]. Thus, vasopressin caused an impairment of renal oxygenation, as demonstrated by an increase in renal oxygen extraction, suggesting that the use of vasopressin in liver transplantation might be a two-edged sword.

One limitation of the present study is the relatively low number of included liver transplant recipients. Furthermore, we did not assess whether or not there was a structural tubular cell injury in the early postoperative period in this group, as we did not measure tubular injury markers. However, the incidence of AKI in the present study was high (67%), when compared to previous studies on tubular injury markers after liver transplantation (38–46%) [28, 29], and it is likely that release of tubular injury markers occurred also in the present study. The strength of the present study is that it provides new information on renal function and oxygenation in the early period after liver transplantation.

## Conclusions

There is a substantial decline in renal function early after liver transplantation despite hyperdynamic circulation and renal vasodilation. This early decline in renal function is accompanied by an impaired renal oxygenation, as the pronounced elevation of renal oxygen consumption is not met by a proportional increase in renal oxygen delivery.

## Key messages

After liver transplantation:

- there is an early and substantial fall in GFR caused by a vasodilation of the efferent arterioles
- renal oxygen consumption is considerably increased
- renal oxygen delivery does not meet the increased renal metabolic demand despite renal vasodilation
- renal oxygenation is impaired

## Abbreviations

[Na^+^]_s_: Serum sodium concentration
[Na^+^]_u_: Urine sodium concentration
^51^Cr-EDTA: Chromium ethylenediaminetetraaceticacid
ACE inhibitor: Angiotensin-converting enzyme inhibitor
AKI: Acute kidney injury
ASA: American Society of Anesthesiologists
CaO_2_: Arterial oxygen content
CO: Cardiac output
CrvO_2_: Renal vein oxygen content
CVP: Central venous pressure
GFR: Glomerular filtration rate
HRS: Hepatorenal syndrome
ICU: Intensive care unit
MAP: Mean arterial pressure
MDRD: Modification of Diet in Renal Disease
MELD: Model For End-Stage Liver Disease
mGFR: Measured glomerular filtration rate
RBF: Renal blood flow
SVI: Stroke volume index
SVRI: Systemic vascular resistance index
